# Hyperoxia as a driver of gut dysbiosis

**DOI:** 10.3389/fmicb.2025.1675652

**Published:** 2025-11-13

**Authors:** Hang Wu, Wenxia Zeng, Ninan Dai, Juan Gu, Ying He, Han Qin, Long Lin, Xiaoyun Fu, Bao Fu, Zhouxiong Xing

**Affiliations:** 1 Department of Critical Care Medicine, Affiliated Hospital of Zunyi Medical University, Zunyi, China; 2 Department of Pharmacy, Affiliated Hospital of Zunyi Medical University, Zunyi, China; 3 Department of Respiratory and Critical Care Medicine, Kweichow Moutai Hospital, Zunyi, China; 4 Department of Nephrology, Kweichow Moutai Hospital, Zunyi, China; 5 Guizhou Provincial Key Laboratory of Medicinal Biotechnology in Colleges and Universities, Affiliated Hospital of Zunyi Medical University, Zunyi, China

**Keywords:** hyperoxia, gut dysbiosis, oxygen therapy, butyrate, reactive oxygen species, electron acceptors

## Abstract

The mammalian colon lumen exists in a highly anaerobic environment (oxygen partial pressure (PO_2_) < 1 mmHg), which promotes the growth of beneficial obligate anaerobes (OA) while limiting the expansion of pathogenic facultative anaerobes (FA). Gut dysbiosis is associated with a wide range of human diseases, and is often characterized by an overgrowth of FA, particularly those in the Enterobacteriaceae family. Oxygen (O_2_) plays a crucial role in bacterial physiology and ecology, and increased O_2_ availability is a key driver of gut dysbiosis. O_2_ therapy is commonly used for hypoxic patients, either through inhalation or extracorporeal membrane oxygenation (ECMO), both of which can expose the gut to excess O_2_, known as hyperoxia. Hyperoxia leads to the overproduction of reactive O_2_ species, resulting in organ injury and worsening clinical outcomes. Viewing gut dysbiosis from an ecological perspective highlights the disruption of host mechanisms that regulate the gut microbiota, particularly in the context of antibiotic use and a western (low fiber) diet, where physiological hypoxia in the colonic epithelium is compromised. This review extends that perspective to O_2_ therapy in acute care, discussing the rationale and experimental evidence linking hyperoxia to gut dysbiosis, with a focus on venoarterial (VA)-ECMO support as a potential contributor. Understanding these mechanisms could help clinicians optimize O_2_ management during therapy.

## Introduction

Oxygen (O_2_) therapy has become one of the most widely prescribed treatments globally since its initial documentation in 1890 (Matthay, 2015; Siemieniuk et al., 2018). However, its toxicity—primarily resulting from the excessive production of reactive oxygen species (ROS)—has always been a major concern. When the inhaled oxygen concentration (FiO₂) exceeds 0.21, it can lead to hyperoxemia (defined as an arterial partial pressure of oxygen PaO₂ > 100 mmHg), causing an excess of oxygen in the blood. This condition triggers hyperoxia, characterized by abnormally elevated tissue oxygen levels, which in turn exacerbates ROS production. Therefore, hyperoxemia is an abnormal indicator in arterial blood, while hyperoxia is its subsequent manifestation at the tissue level, with the latter directly linked to oxidative damage (Singer et al., 2021). Given the potential risks of hyperoxia, the judicious use of oxygen in clinical practice has become a critical issue. In the acute hospital setting, particularly in emergency, respiratory critical care, cardiac, and anesthesiology departments, oxygen therapy is a cornerstone of nursing care (Gelissen et al., 2021; Schjørring et al., 2021). While O_2_ supplementation is lifesaving for patients experiencing respiratory and/or circulatory failure, super-physiological levels of O_2_-referred to as hyperoxia- can lead to the overproduction of reactive O_2_ species (ROS), causing harmful effects both systemically and locally and poor clinical outcomes (Damiani et al., 2018; Nolfi-Donegan et al., 2020). O_2_ can be delivered to patients by a variety of techniques, ranging from the simple O_2_ therapy (inhalation) to complex extracorporeal membrane oxygenation (ECMO) (Gu et al., 2017). Traditionally, hyperoxic toxicity during O_2_ inhalation (e.g., via mechanical ventilators), has been a concern particularly to the lung (Hochberg et al., 2021; Singer et al., 2021). However, the increasing use of venoarterial (VA) -ECMO for patients with severe cardiac or cardiopulmonary failure challenges this view (Winiszewski et al., 2022; Dai et al., 2024). VA-ECMO introduces lower-body hyperoxia during dual circulation, which differs from the typical alveolar hyperoxia seen with O_2_ inhalation and can also have significant effects on the gastrointestinal tract (Winiszewski et al., 2018; Asija et al., 2023; Winiszewski et al., 2024).

The human gut is home to trillions of microbes, the majority of which reside in the colon, an environment characterized by extremely low O_2_ levels (with a partial pressure of O_2_ (PO_2_) less than 1 mmHg) (Human Microbiome Project Consortium, 2012; Albenberg et al., 2014). The colonic epithelium normally exists in a state of physiological hypoxia that is crucial for maintaining gut homeostasis by supporting the anaerobic conditions (anaerobiosis) in the lumen (Cummins and Crean, 2017; Wang et al., 2024). The predominant microbes in the colon are OA that produce beneficial short fatty chain acid (SCFA), primarily belonging to the *Clostridia* (*Phylum Firmicutes*) and *Bacteroidia* (*Phylum Bacteroidetes*) classes, which together make up over 90% of the microbial population (Rivera-Chávez et al., 2017). The limited availability of external respiratory electron acceptors, such as O_2_, restricts the growth of FA, including those from the *Bacilli* class (*Phylum Firmicutes*) and the *Enterobacteriaceae* family (*Phylum Proteobacteria*), which represent the leading sources of pathogenic microorganisms in clinical practices and only a small faction (<5%) of the overall microbial community in a healthy gut (Eckburg et al., 2005). Dysbiosis, or an imbalance in microbial community, typically refers to an overgrowth of potentially harmful FA (biomarker: *Enterobacteriaceae*) or a reduction in beneficial OA (Chanderraj et al., 2023). This imbalance has been linked to various chronic and acute diseases in humans and is associated with poorer clinical outcomes (Dickson, 2016; Lynch and Pedersen, 2016; Winter and Bäumler, 2023). Over the past decade, an ecological perspective has highlighted O_2_ as a critical factor driving gut dysbiosis, particularly in the context of the interactions between gut microbes and the host (Rivera-Chávez et al., 2017; Litvak and Bäumler, 2019; Lee et al., 2022). This review synthesizes current evidence, revisits established concepts, and discusses how hyperoxia, especially from VA-ECMO support, contributes to gut dysbiosis by disrupting the “OA-fiber-butyrate-colonocyte metabolism (oxidative phosphorylation) -epithelial physiological hypoxia-luminal anaerobiosis” axis.

## O_2_ and microbial physiology, ecology and evolution

O_2_, a diatomic molecule, contains two unpaired electrons in its atomic structure, giving it high reactivity potential (Hara and Kondo, 2015). Owing to this high reactivity, O_2_ is pivotal in bacterial physiology, ecology and evolution by driving energy metabolism and supporting the synthesis of essential polymers for cell growth and population expansion (Fenchel and Finlay, 2008; Shan et al., 2012).

**Figure 1:**
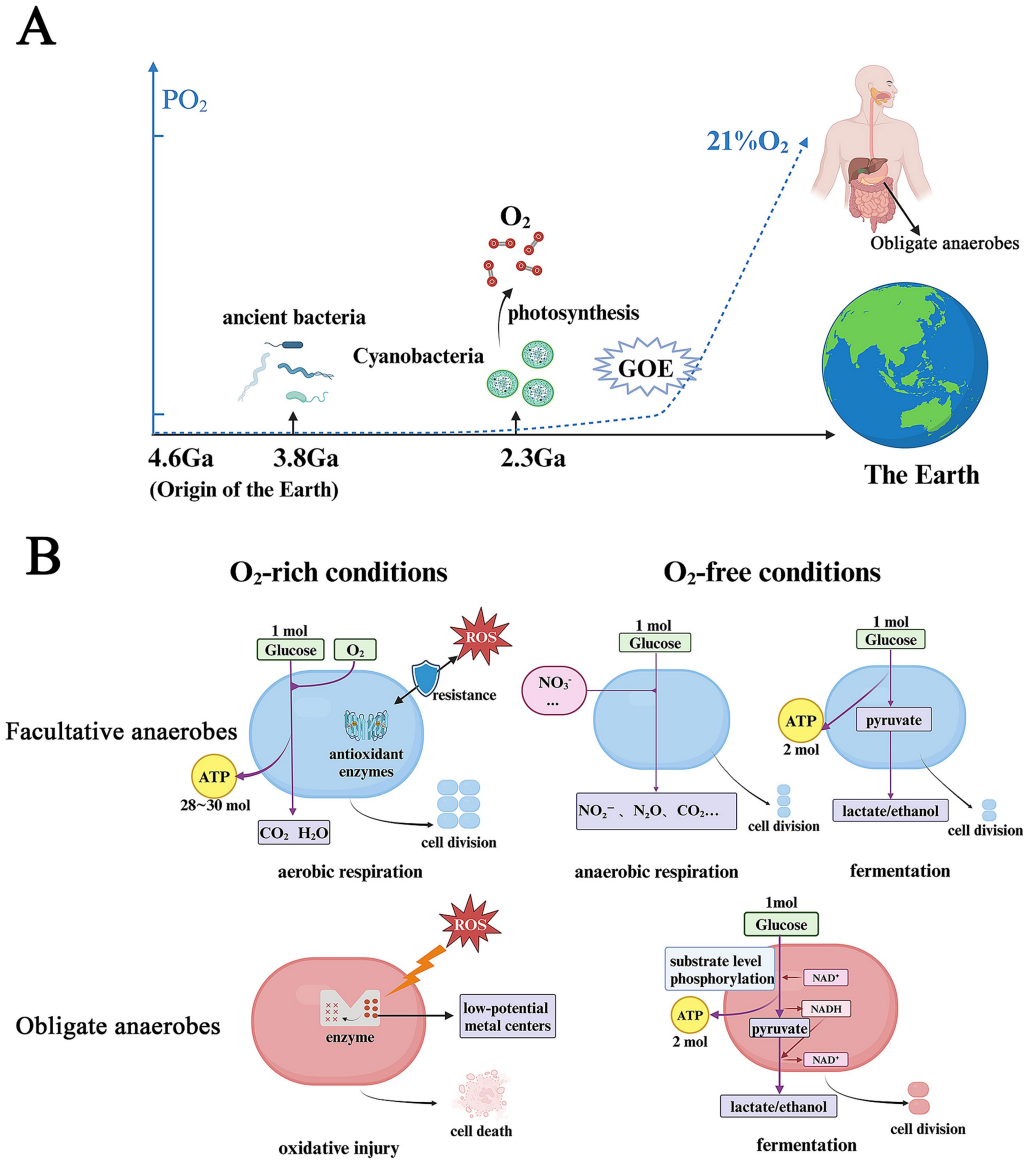
O_2_ plays a central role in co-evolution of the Earth and microbes and microbial metabolic reprogramming. **(A)** O_2_ drives the co-evolution of the Earth and microbes. **(B)** Bacterial metabolic reprogramming in O_2_-free and -rich conditions. PO_2_, partial pressure of oxygen; Ga, giga-annum; GOE, great oxidation event; ROS, reactive oxygen species. Figure created with BioRender.com.

Earth’s early atmosphere and ocean were extremely anoxic, with O_2_ levels less than 10^−5^ of their current concentration. Aerotolerant bacteria are thought to have emerged approximately 3.8 Giga-annum (Ga) ago (Kasting and Siefert, 2002; Lyons et al., 2014). The accumulation of O_2_ began after the advent of oxygenic photosynthesis in Cyanobacteria around 2.3 Ga (Payne et al., 2011; Fischer and Valentine, 2019). This process led to a significant increase in atmospheric O_2_ levels, known as the Great Oxygenation Event (GOE), which paved the way for the evolution of complex organisms, including animals, that relied on O_2_ as a high-potential electron acceptor for producing adenosine triphosphate (ATP) (Olejarz et al., 2021). ATP, known as leading energy currency in cellular processes, drives metabolic activities, protein synthesis and microbial bioproduction (Hara and Kondo, 2015; Nolfi-Donegan et al., 2020). Under selective pressure, the anaerobic respiratory chains of certain bacteria such as FA, adapted to use O_2_ as a new terminal electron acceptor. Meanwhile, FA rely on a complex antioxidant enzyme network composed of superoxide dismutase (SOD), catalase, peroxidase, and the thioredoxin system. They have evolved crucial antioxidant enzymes to counteract the toxicity of oxygen, enabling successful colonization across ecological niches with varying oxygen conditions (Raymond and Segrè, 2006; Johnson and Hug, 2019; Ślesak et al., 2019). A different group of microorganisms, such as OA, continued their anaerobic metabolic processes and adapted to O_2_-free habitats, including hot springs, lake sediments and the human gut (Lu and Imlay, 2021). These findings highlight the intertwined evolution of microbial life and earth’s early environments as the planet transitioned from an O_2_-deficient to an O_2_-rich atmosphere (Lyons et al., 2024) (Figure 1A).

Under O_2_-rich conditions, O_2_ inhibits the growth of OA that lack the mechanisms to defend against ROS. This vulnerability arises because anaerobic metabolism relies on catalytic sites with low-potential metal centers, which are highly susceptible to damage from ROS (Lu and Imlay, 2021). In contrast, FA utilize O_2_ as an external terminal electron acceptor during aerobic respiration (oxidative phosphorylation). This process produces approximately 28–30 molecules of ATP per molecule of glucose, supporting a significant increase in biomass production. Under anoxic conditions, OA primarily rely on fermentation, a process in which nicotinamide adenine dinucleotide (NADH) is oxidized by endogenous intermediates (electron acceptors) produced from the decomposition of carbon sources. ATP is generated solely through substrate- level phosphorylation, a process known as glycolysis (2 ATP molecules per glucose molecule metabolized into lactate) (Koropatkin et al., 2012; Shan et al., 2012). In response to O_2_ scarcity, FA undergo substantial metabolic reprogramming, shifting from the aerobic respiration to anaerobic respiration using alternative exogenous electron acceptors (such as nitrate) or fermentation (Bueno et al., 2012; Shan et al., 2012) (Figure 1B).

Thus, microbes using the redox reaction yielding the greatest ATP production prevail, during which availability to exogenous electron acceptors (especially O_2_) determines which metabolic groups of bacteria can dominate the microbial communities in an ecological nutrient-niche (Lee et al., 2022).

## Coordinated control of multiple factors in the GI microenvironment

The composition and function of the gut microbiota are influenced by oxygen. However, this principle does not fully capture the reality within the specific microenvironment of the human gastrointestinal tract. The gut microbiota is also co-regulated by a variety of biochemical and physiological factors, including pH, antimicrobial peptide (AMP) distribution, bile acids, and intestinal transit time.

When it comes to pH, numerous studies in murine models have demonstrated that the pH gradient along the gastrointestinal tract—from the highly acidic environment of the stomach to the near-neutral conditions in the colon—significantly regulates the structure and function of the gut microbiota. For example, in these models, a lower pH environment (e.g., pH 5.5) generally favors the growth of certain members of the Firmicutes phylum and Bifidobacterium, and promotes butyrate production. In contrast, under higher pH conditions (e.g., pH 6.5–7.0), Bacteroidetes members tend to be more active. pH not only directly influences microbial growth and enzyme activity but also indirectly affects intestinal environmental stability and microbial community structure by modulating the production and absorption of metabolites such as short-chain fatty acids (SCFAs) (Yamamura et al., 2023; Xie et al., 2024). Secondly, bile acids are important molecules synthesized by the liver and metabolized by gut microorganisms, playing a central regulatory role in the intestinal microbiota. Research using mouse models has shown that they disrupt microbial membrane structures through surfactant activity and exert selective antibacterial effects by inhibiting the growth of certain bacteria while promoting the colonization of tolerant species. Meanwhile, microorganisms modify bile acids via enzymes such as bile salt hydrolases, altering their signaling activity and toxicity, thereby modulating the structure of the microbiota and the host’s immune responses (Guzior and Quinn, 2021; Kim et al., 2024). Meanwhile, studies in fish models have demonstrated that dietary AMP supplementation significantly reshaped the gut microbiota structure. Antimicrobial peptides (AMPs) exhibit a dose-dependent “double-edged sword” effect. At appropriate doses, they optimize microbial composition (e.g., promoting Firmicutes while suppressing Bacteroidetes), improve nutrient metabolism, and maintain beneficial microbial stability under pathogenic stress, thereby enhancing ecological resilience. However, excessive supplementation may increase the proportion of Proteobacteria, consequently disrupting microbial homeostasis (Liu et al., 2022). However, in addition to the aforementioned chemical factors, gut transit time—as a core physio-mechanical variable—exhibits a tight bidirectional interaction with the microbiome. Human studies have indicated that it not only serves as a key driver of microbial composition and metabolism (such as the balance between glycolysis and proteolysis), but is itself modulated by microbial metabolites (Procházková et al., 2023).

Therefore, in addition to oxygen, factors such as intestinal pH, the antimicrobial peptide (AMP) gradient, the presence of bile acids, and transit rate are also crucial for the microbiome.

## Gastrointestinal O_2_ gradient shapes microbial composition

**Figure 2:**
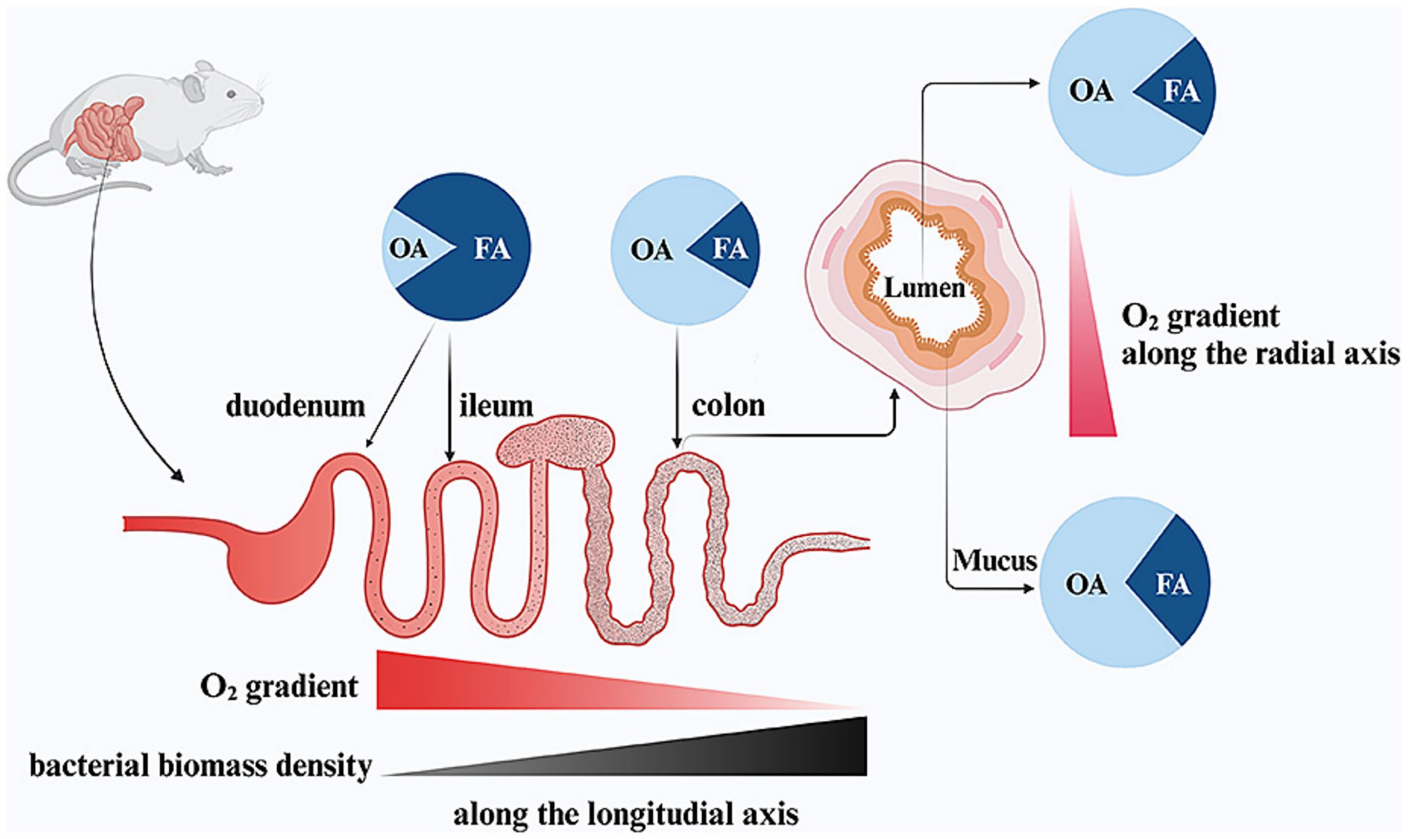
The effect of intestinal oxygen gradients on the gut microbial composition in mice. OA, obligate anaerobes, FA, facultative anaerobes. Figure created with BioRender.com.

At sea level, the PO_2_ in breathable air is approximately 150 mmHg (21% O_2_), which leads to a PO_2_ of 100–110 mmHg (15% O_2_) within healthy human alveoli, an arterial PO_2_ (PaO_2_) of 90–100 mmHg (12% O_2_) and a PO_2_ of 60–70 mmHg (8% O_2_) in the human liver (Schaible et al., 2010; Singhal and Shah, 2020). In contrast, studies in mice show that the intestinal mucosa exists in a relatively low-PO_2_ environment, showing a steep O_2_ gradient both along the length of the intestine (longitudinal axis) and from the inner lumen to the outer serosa (radial axis) (Zheng et al., 2015). In the murine gut, O_2_ levels in the gut lumen remarkably decline as intestinal contents move from the upper to the lower digestive tracts (Friedman et al., 2018). In the stomach and duodenum, O_2_ levels in tissues and the lumen are similar. However, starting in the ileum, these levels diverge significantly. The luminal O_2_ concentration drops sharply, reaching its lowest point in the colon (<1 mmHg, 0.2% O_2_) (Singhal and Shah, 2020). The murine host maintains the colonic epithelium in a physiological hypoxic state (<10 mmHg, 2% O_2_) due to mitochondrial O_2_ consumption through oxidative phosphorylation, which is coupled with oxidation of fatty acids (Litvak et al., 2018; Zhang et al., 2022). This mechanism restricts the diffusion of O_2_ into the intestinal lumen, creating anaerobosis (Pral et al., 2021). Pure O_2_ inhalation in mouse models results in hyperoxia, leading to a rise in luminal PO_2_. This increase confirms that O_2_ diffuses from intestinal tissues and forms a radial gradient extending from the mucosal tissue interface into the lumen (Albenberg et al., 2014). Both the longitudinal and radial O_2_ gradients within the gut play a critical role in determining the composition of the gut microbiota (Donaldson et al., 2016; Miller et al., 2021) (Figure 2).

Research in mice demonstrates that high O_2_ tension in the small intestine favors the growth of aerotolerant bacteria (Gu et al., 2013; Sundin et al., 2017). In contrast, the near-zero O_2_ levels in the colon lumen support the growth of organisms which cannot tolerate O_2_ (Lee et al., 2022). The luminal O_2_ concentrations create a distinct environment that shapes the composition of the gut microbiota. As a result, the small intestine in mice is primarily colonized by FA, such as members of the *Enterobacteriaceae* family (*Phylum Proteobacteria*) and *Lactobacillaceae* family (*Phylum Firmicutes*) (Gu et al., 2013; Sundin et al., 2017). Meanwhile, the colon is dominated by OA, including classes such as *Bacteroidia* (*Phylum Bacteroidetes*) and *Clostridia* (*Phylum Firmicutes*) (Rivera-Chávez et al., 2017). Similarly, aligning with the radial O_2_ gradient, FA such as *Proteobacteria* and *Actinobacteria* are more prevalent in the mucosal interface (mucus layer) compared to the lumen (Albenberg et al., 2014; Friedman et al., 2018). At the same time, the bacteria biomass undergoes a dramatic increase, rising by more than a million-fold from the small intestine (10^3^–10^5^ cells/ml) to the colon (10^11^–10^12^ cells/ml), which corresponds to the decreasing O_2_ levels in the lumen (Sundin et al., 2017; Friedman et al., 2018; de Vos et al., 2022).

In infancy, there is a sequential colonization by aerotolerant bacteria, followed by a transition to anaerobic bacteria during childhood and adulthood (Orrhage and Nord, 1999). It was once believed that FA were responsible for creating the O_2_ gradient by progressively consuming O_2_ as it moves through the small intestine, thus preserving colonic anaerobiosis (Adlerberth and Wold, 2009). However, key experiments comparing conventional and germ-free mice have shown that similar PO_2_ values are observed in corresponding gut segments of both conventional and germ-free mice (Friedman et al., 2018). Evidence indicates that the steep O_2_ gradient along the gastrointestinal tract is primarily driven by the host rather than microbial O_2_ consumption (Lee et al., 2022). Taken together, the host-controlled anaerobiosis in the colon supports the growth of trillions of OA while restricting the expansion of pathogenic FA.

## Physiology of O_2_ therapy

O_2_ therapy is the standard treatment for hypoxic patients with cardiopulmonary diseases, aiming to ensure adequate tissue oxygenation (Chiumello and Brioni, 2016; Vieira et al., 2020). Tissue hypoxia typically results from an imbalance between O_2_ delivery (DO_2_) and O_2_ consumption (VO_2_) in circulation. In resting adults, DO_2_ and VO_2_ are approximately 600 mL/min/m^2^ and 120 mL/min/m2, respectively (Pinsky, 2007). Under normal physiological conditions, the DO_2_: VO_2_ ratio is maintained at 5:1. When this ratio falls below 2:1, multiple organ dysfunction syndrome (MODS) and lactic acidosis may occur (Lim, 2023). DO_2_ is determined by the patient’s cardiac output (CO) and the arterial O_2_ content (CaO_2_), as described by the following formula (Wemple et al., 2023a,b):


$${\mathrm{DO}}_{2}=\mathrm{CO}\times {\mathrm{CaO}}_{2}$$

The CaO_2_ is determined by the product of hemoglobin (Hb), O_2_ saturation (SaO_2_), and Hufner’s constant (typically 1.36 mL/g), along with arterial dissolved O_2_, which is considered negligible due to O_2_’s low solubility. Therefore, the calculation of CaO_2_ can be simplified using the following formula (Lim, 2023):


$${\mathrm{CaO}}_{2}=\mathrm{Hb}\times {\mathrm{SaO}}_{2}\times 1.36\;\mathrm{mL}/g+0.0031\times {\mathrm{PaO}}_{2}$$

$$\approx \mathrm{Hb}\times {\mathrm{SaO}}_{2}\times 1.36\;\mathrm{mL}/g$$

SaO_2_ depends on arterial PO_2_ (PaO_2_) due to the sigmoidal shape of the O_2_ dissociation curve (Collins et al., 2015). Hence, tissue hypoxia may results from decreased CO and/or decreased PaO_2_, which are fundamental physiological changes during cardiac failure and/ or pulmonary failure in patients.

O_2_ delivery systems used in clinical settings can be categorized into O_2_ inhalation and ECMO, each with distinct indications and physiological mechanisms (Lustbader and Fein, 2000). It is also important to differentiate between hyperoxemia and hyperoxia. Hyperoxemia is defined as PaO_2_ > 100 mmHg, while hyperoxia refers to excessive O_2_ at the cellular level (Singer et al., 2021). O_2_ inhalation, also known as traditional O_2_ therapy, remains the primary method for treating hypoxic patients with mild to moderate respiratory failure, such as mild acute respiratory distress syndrome (ARDS) (Angus, 2020). However, ECMO is more commonly used for patients with severe pulmonary or cardiopulmonary failure, including severe ARDS, refractory cardiogenic shock, and cardiac arrest (Chiumello and Brioni, 2016; Combes et al., 2020). Inhaled O_2_ is administered to the patient through the upper respiratory tract using specialized devises (e.g., mechanical ventilators), which increase the fraction of inspired O_2_ (FiO_2_) as well as the partial pressure of alveolar O_2_ (PAO2). The elevated PAO_2_ drives O_2_ diffusion across the alveolar-capillary barrier and increases PaO_2_, thereby increasing DO_2_ and improving oxygenation (Wemple et al., 2023a,b).

**Figure 3:**
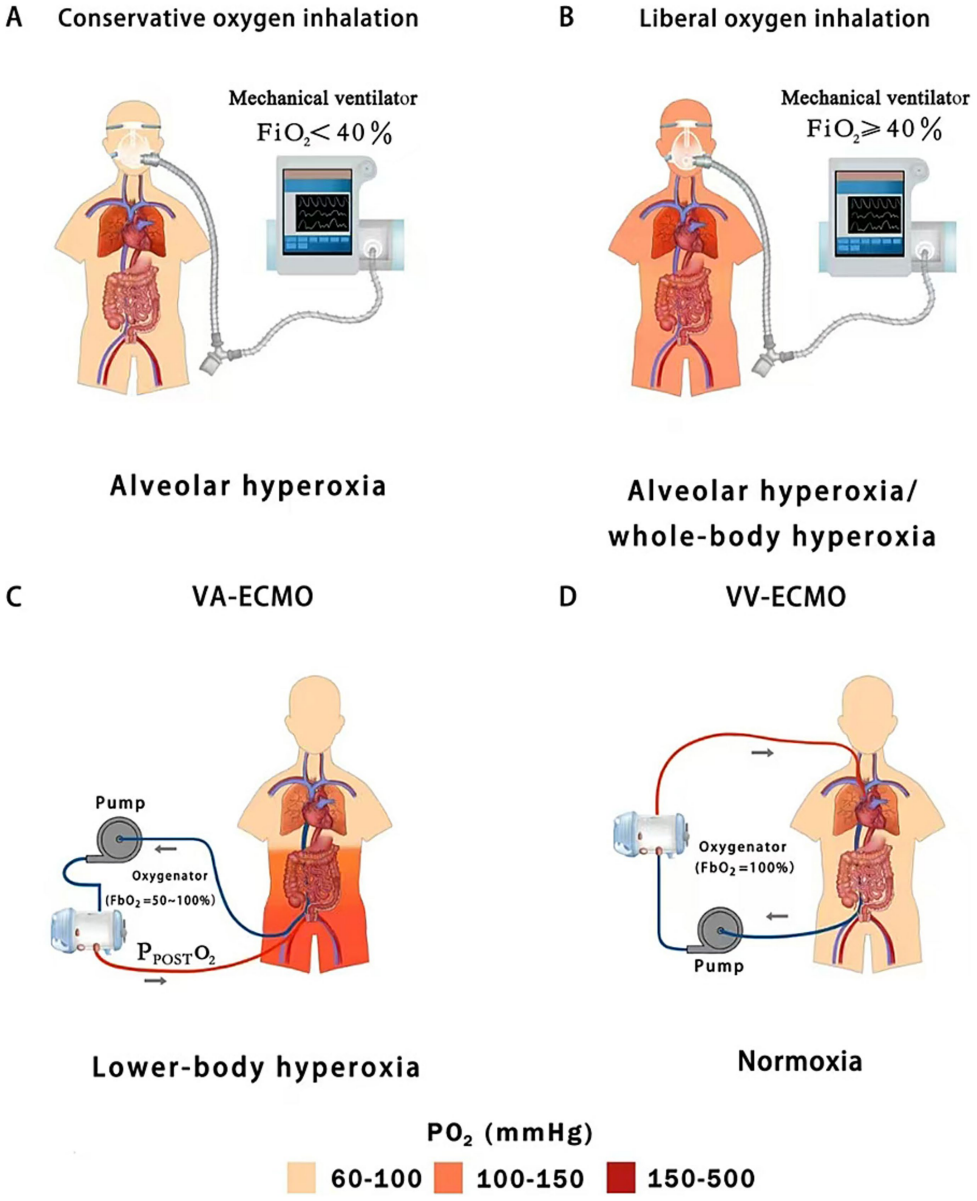
Hyperoxic subtypes of oxygen inhalation (conservative vs. liberal oxygen therapy) and peripheral extracorporeal membrane oxygenation (VA vs. VV); **(A)** Conservative oxygen therapy can cause slightly high alveolar oxygen concentrations; **(B)** Liberal oxygen therapy leads to moderate or severe alveolar hyperoxia and mild whole-body hyperoxia; **(C)** Peripheral VA-ECMO may lead to moderate or severe lower-body hyperoxia; **(D)** Peripheral VV-ECMO usually does not cause hyperoxemia; FiO_2_, fraction of inspired oxygen; P_POST_O_2_, post-oxygenator oxygen partial pressure; FbO_2_, fraction of inspired O_2_ of blender; VA-ECMO, venoarterial-extracorporeal membrane oxygenation; VV-ECMO, venovenous-extracorporeal membrane oxygenation.

ECMO can be initiated in two distinct forms: venovenous (VV) or venoarterial (VA). VV-ECMO primarily supports the respiratory system, while VA-ECMO supports both the cardiac and respiratory systems simultaneously. Desaturated blood is drawn from the inferior vena cava by a centrifugal pump, and oxygenation occurs in an external membrane oxygenator, and finally the oxygenated blood is directly delivered into either the venous system (VV-ECMO) to increase mixed venous O_2_ pressure (PVO_2_) and PaO_2_ or the arterial system (VA-ECMO) to provide additional CO (Vieira et al., 2020). In this process, O_2_ input levels are regulated by the fraction of inspired O_2_ of blender (FbO_2_) supplied to the oxygenator (Winiszewski et al., 2022) (Figure 3).

## Intestinal hyperoxia during O_2_ therapy

O_2_ inhalation and ECMO expose different organ systems to the risk of hyperoxia (Winiszewski et al., 2022; Dai et al., 2024). O_2_ inhalation is typically administered using either a liberal or conservative strategy, targeting either a high level (FiO_2_ ≥ 40%, PaO_2_ = 100–150 mmHg) or a low level (FiO_2_ < 40%, PaO_2_ = 70–100 mmHg) of oxygenation, respectively (Girardis et al., 2016). Conservative O_2_ inhalation reduces O_2_ toxicity by preventing arterial hyperoxemia (whole-body hyperoxia) and lowering the risk of alveolar hyperoxia (P_A_O_2_ < 300 mmHg). In contrast, patients receiving liberal O_2_ inhalation face a higher risk of alveolar hyperoxia (P_A_O_2_ ≥ 300 mmHg) and whole-body hyperoxia, including the gut (Wemple et al., 2023a,b; Dai et al., 2024) (Figures 3A,B).

VA-ECMO is increasingly used to provide circulatory support in patients with severe pump failure (low CO) by delivering additional oxygenated blood and improving DO_2_. VA-ECMO is characterized by dual circulation, during which competitive flow develops between blood ejected from the native heart and oxygenated blood traveling retrograde within the aorta from the ECMO reinfusion cannula, which is inserted into the femoral artery (Asija et al., 2023). As a result, oxygenation of the lower body is determined by the fraction of FbO_2_ and post-oxygenator partial pressure of O_2_ (P_POST_O_2_). In current clinical practice, FbO_2_ is typically set between 50 and 100%, leading to a P_POST_O_2_ of 300–500 mmHg at the membrane lung outlet. This directly exposes intra-abdominal organs, especially the gastrointestinal tract and its resident microbial communities to severe hyperoxia (Winiszewski et al., 2022; Jentzer et al., 2023; Premraj et al., 2023; Dai et al., 2024) (Figure 3C). Blood flow to the large intestine is mainly supplied by the superior and inferior mesenteric arteries, which are located near the outlet of the VA-ECMO reinfusion cannula (Winiszewski et al., 2018; Shetty et al., 2024). As a result, the colon is directly perfused by hyperoxia blood during VA-ECMO support, raising the levels of O_2_ diffused from the colonic vasculature into the lumen and disrupting the anaerobic environment.

VV-ECMO oxygenates the venous blood outside the body, increasing PvO_2_ in the right atrium, thereby increasing PaO_2_ and DO_2_. During VV-ECMO support (e.g., using femoro-jugular VV bypass), venous blood (the inferior Vena Cava) is drawn into the ECMO circuit depending on the ECMO flow relative to the total venous return (equivalent to CO), while a proportion of venous deoxygenated blood (the Superior Vena Cava) directly returns to the right heart bypassing the ECMO circuit. The admixture of ECMO-oxygenated blood and deoxygenated venous blood results in an increased PvO_2_ (e.g., 60–80 mmHg) rather than hyperoxia (PO_2_ > 100 mmHg) in the pulmonary circulation (Walker et al., 2007; Lim, 2023). In fact, patients with severe ARDS are often still hypoxic or normoxic despite full ECMO support (FbO_2_ = 100%) (Montisci et al., 2015). Therefore, VV-ECMO rarely leads to hyperoxia on its own due to the shunting of deoxygenated venous blood (Lim, 2023) (Figure 3D). In summary, intestinal hyperoxic injury remains a concern particularly in the settings of VA-ECMO (Winiszewski et al., 2018).

However, studies in rat models have shown that hyperoxia exposure can also significantly impair intestinal barrier function. Hyperoxia induces excessive production of reactive oxygen species (ROS) in the body, leading to the downregulation of key intestinal tight junction proteins such as ZO-1, Occludin, and Claudin-4. This disrupts the intercellular connections in the intestinal epithelium, resulting in the loss of intestinal barrier integrity and increased permeability. Consequently, bacteria and their metabolites, such as D-lactic acid and endotoxins, can translocate into the bloodstream. This further triggers an imbalance in local intestinal inflammatory factors, creating a vicious cycle (Liu et al., 2020).

## Hyperoxia is associated with poor clinical outcomes

Inhaling O_2_ (FiO_2_ > 0.21) can lead to arterial hyperoxemia (PaO_2_ > 100 mmHg) and, consequently, tissue hyperoxia, which may increase the production of ROS (Singer et al., 2021). Clinical studies have shown that severe hyperoxemia (PaO_2_ > 300 mmHg) during O_2_ inhalation is an independent predictor of higher in-hospital mortality in critically ill patients (Kilgannon et al., 2010, 2011; Rincon et al., 2014; Singer et al., 2021). In human patients, administering inhaled O_2_ to patients without hypoxia has been associated with increased mortality without improving clinical outcomes (Chu et al., 2018). Based on these evidences, more recent clinical guidelines advise a more cautious approach to O_2_ inhalation to reduce its potential toxic effects (O’Driscoll et al., 2017; Siemieniuk et al., 2018). A target peripheral capillary O_2_ saturation of ≤ 96% (PaO_2_ ≤ 100 mmHg) is generally recommended for most acutely ill patients (Siemieniuk et al., 2018). Over the past decade, many clinicians have adjusted O_2_ levels (FiO_2_ and PaO_2_) more carefully to prevent severe hyperoxia in clinical practice (Damiani et al., 2018; Mackle et al., 2020; Hochberg et al., 2021).

Several studies in patients examining hyperoxia in the context of VA-ECMO have reported a link between hyperoxia and adverse clinical outcomes (Rao et al., 2018; Winiszewski et al., 2022; Tigano et al., 2023; Winiszewski et al., 2024). A recent meta-analysis of clinical data revealed that severe hyperoxia following the initiation of VA-ECMO was associated with a twofold increase in rates of poor neurological outcomes and mortality (Tigano et al., 2023). Current guidelines from the Extracorporeal Life Support Organization recommend adjusting F_S_O_2_ to achieve mild hyperoxia (P_POST_O_2_ = 150 mmHg) in order to prevent both excessive hypo- and hyperoxemia (Winiszewski et al., 2022). Despite this, moderate (PaO_2_ = 150–300 mmHg) and severe hyperoxemia (PaO_2_ > 300 mmHg) are frequently observed during VA-ECMO support, affecting approximately 30 and 20% of patients, respectively (Rao et al., 2018). P_POST_O_2_, which serves as an indicator of the lower body and intestinal oxygenation, is rarely measured in clinical settings, yet when it is, it often shows significant hyperoxemia with a median value nearing 200 mmHg (Winiszewski et al., 2024). Additionally, the median F_S_O_2_ value was found to be 70%, with a higher F_S_O_2_ levels independently associated with increased mortality (Winiszewski et al., 2024). These findings point to a strong connection between hyperoxia, as a result of VA-ECMO blood flow, and poor clinical outcomes.

## Gut dysbiosis is associated with poor clinical outcomes

Advancements in culture-independent technologies, such as metagenomics and metabolomics, have led to a deeper understanding of the complex interactions between human health, disease, and the gut microbiota (Lynch and Pedersen, 2016; de Vos et al., 2022). It is increasingly recognized that an imbalance in the gut microbiome, known as dysbiosis, is linked to a wide range of chronic diseases, including obesity (Cani and Jordan, 2018), diabetes (Qin et al., 2012; Ceccarani et al., 2020), chronic kidney disease (Hida et al., 1996), cardiovascular disease (Jie et al., 2017), inflammatory bowel disease (Frank et al., 2007) and colorectal cancer (Louis et al., 2014). Dysbiosis has also been linked to acute conditions like sepsis (Haak and Wiersinga, 2017; Adelman et al., 2020), acute pancreatitis (Ammer-Herrmenau et al., 2024), stroke (Peh et al., 2022), intracerebral hemorrhage (Xu et al., 2019), pneumonia and acute respiratory distress syndrome (Dickson, 2016). In many of these conditions, gut dysbiosis is characterized by a shift in the microbial community OA (health-promoting microbes) to FA (pathobiota) (Corriero et al., 2022; Winter and Bäumler, 2023; Li et al., 2024).

## Increased availability of electron acceptors (O_2_) drives gut dysbiosis

Recently, ecological and evolutionary theories from macroecology have been increasingly used to explain phenomena related to the microbes and their host (Berg et al., 2020; McDonald et al., 2020). The gut microbiome refers to an environment and the community of microbes within it, while gut dysbiosis is characterized not only by abnormal microbial compositions, functions and metabolites but also by disruptions in both microbe-microbe and microbe-host interactions (Tipton et al., 2019; Ashley et al., 2020).

**Figure 4:**
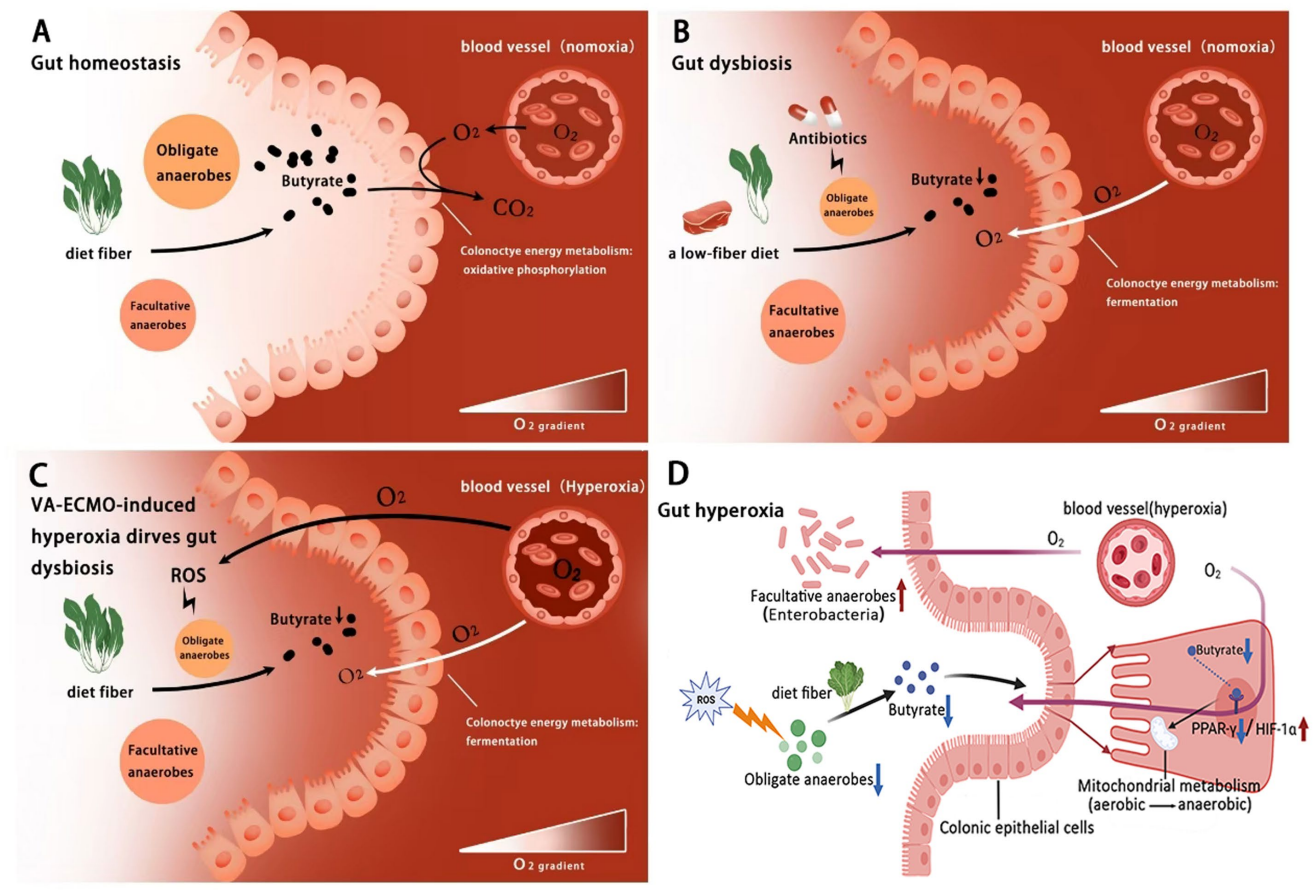
Hyperoxia during VA-ECMO as a driver of gut dysbiosis. **(A)** Under normal gut homeostasis, the limited availability of external respiratory electron acceptors (O_2_) in the colon lumen restricts the growth of facultative anaerobes, maintaining a balanced gut microbial community; **(B)** In cases of gut dysbiosis, factors such as antibiotic use and a low-fiber diet disrupt this balance by increasing O_2_ availability in the colon lumen, promoting microbial imbalance; **(C)** VA-ECMO-induced hyperoxia leads to both primary and secondary increases in O_2_ availability in the colonic lumen, driving gut dysbiosis. **(D)** Butyric acid may regulate hyperoxia-Induced energy metabolism disorder in colonic epithelium via the PPAR-*γ*/HIF-1α pathway. ROS, reactive O_2_ species.

In the healthy gut, the colon lumen is dominated by commensal OA which converts resistant starch and dietary fiber (nonstarch polysaccharides) into SCFA (mainly acetate, propionate, and butyrate) through saccharolytic fermentation (Morrison and Preston, 2016; Louis and Flint, 2017). SCFA contributes to normal colonic function in the humans (Topping and Clifton, 2001). Particularly, butyrate is a preferred substrate for colonocytes which activates nuclear receptor peroxisome proliferator–activated receptor gamma (PPAR-*γ*) and regulates the energy metabolism by switching to *β*-oxidation of fatty acids (Byndloss et al., 2017; Fang et al., 2021). The resulting increase in oxidation of fatty acids in the mitochondria leads to high epithelial O_2_ consumption and maintains the colonic epithelium in a state of physiological hypoxia (Taylor and Colgan, 2017; Singhal and Shah, 2020). Epithelial hypoxia hampers diffusion of O_2_ from vascular capillaries into the colonic lumen, thereby preserving anaerobiosis suitable for OA (Maslowski, 2019). Meanwhile, the paucity of O_2_ as exogenous respiratory electron acceptors for cellular respiration limits the expansion of pathogenic FA (Winter et al., 2013; Bäumler and Sperandio, 2016). During gut homeostasis, the host controls the availability of O_2_ as an important ecological driver governing microbial growth (Byndloss and Bäumler, 2018; Winter and Bäumler, 2023) (Figure 4A).

Consequently, any host factors that increase O_2_ availability in the colonic lumen can disrupt the balance of the gut microbial community (Lee et al., 2024). Studies in murine models have provided key evidence for this mechanism, for instance, antibiotic treatments play a central role in infectious diseases, while they tend to deplete OA from the *Clostridia* class, which are key producers of SCFA, leading to a decrease in butyrate levels in the colon (Garner et al., 2009; Hays et al., 2024). Diet is another important contributor to gut homeostasis which has a profound impact on the composition, function and diversity of the gut microbes (Trakman et al., 2022; Ross et al., 2024). An unhealthy western diet (high-fat low-fiber and high-calorie) leads to a growing prevalence of obesity, diabetes, cardiovascular diseases and cancer (Swinburn et al., 2011; Song et al., 2015). Lack of dietary fiber reduces the fermentation and expression of butyrate by OA in the colon (O’Keefe, 2016; Amabebe et al., 2020). Crucially, experimental evidence from mice has shown that the depletion of butyrate during antibiotic treatment and low-fiber diet shifts the metabolism of colon epithelium cells from oxidative phosphorylation toward fermentation, lowering mitochondrial O_2_ consumption and allowing for greater O_2_ accumulation in the lumen (Kelly et al., 2015; Yoo et al., 2021). As the most efficient terminal electron acceptor in cellular aerobic respiration, O_2_ plays a critical role in energy conservation whose redox activity is mediated by these unpaired electrons as radicals. O_2_ can undergo partial reduction by accepting one, two or three electrons, resulting in the production of ROS such as superoxide (O_2_-) and hydrogen peroxide (H_2_O_2_) (Freinbichler et al., 2011). Oxidative injury induced by ROS can lead to a devastating effect on the structure and activity of proteins, and may even lead to bacterial death (Ezraty et al., 2017). This increase in available O_2_ finally promotes the growth of FA, such as *Enterobacteriaceae*, leading to gut dysbiosis (Rivera-Chávez et al., 2016; Sun et al., 2023) (Figure 4B).

## Hyperoxia alters gut microbial compositions in animals

An early study in mice observed that O_2_ levels in the colonic lumen increased following pure O_2_ inhalation, suggesting that O_2_ diffusion from the host tissue into the intestinal lumen (Albenberg et al., 2014). This finding highlights the potential effects of hyperoxia on gut microbiota and recently spurs the launch of further studies to explore how hyperoxia influences gut microbial compositions (Wang et al., 2023; Dai et al., 2024). In these murine models, a short exposure to hyperoxia (72 h, FiO_2_ = 80–90%) significantly reduced the relative abundance of obligate anaerobic *Ruminococcaceae* family (Class: *Clostridia*) in both the cecum and fecal samples (Ashley et al., 2020; Li Y. et al., 2021). Prolonged exposure to hyperoxia (1–2 weeks, FiO_2_ = 80–90%) not only reduced beneficial OA such as *Ruminococcaceae* (Class: Clostridia) and *Muribaculaceae* (Class: *Bacteroidia*) (Cai et al., 2023), but also promoted the growth of pathogenic FA, such as *Staphylococcus* (Abdelgawad et al., 2023) and *Enterobacteriaceae* (Family: *Proteobacteria*) (Li H. et al., 2021; Li Y. et al., 2021; Lo et al., 2021; Chen et al., 2023). Both *Muribaculaceae* and *Ruminococcaceae* are key producers of SCFAs in the gut (Li H. et al., 2021; Li Y. et al., 2021) and *Muribaculaceae* is particularly abundant in the healthy mouse gut, often comprising 20–30% of the microbial community (Lagkouvardos et al., 2019; Zhu et al., 2024). Further metagenomic analyzes of these mouse models have shown that the depletion of these OA impairs the gut’s ability to produce SCFA by fermentation of dietary fibers, resulting in low levels of butyrate (Cai et al., 2023). Additionally, gut dysbiosis caused by hyperoxia in mice may also contribute to distant organ injury, such as damage to the lungs and brain through the gut-lung (Ashley et al., 2020; Wedgwood et al., 2020; Shen et al., 2024) and gut–brain axes (Lo et al., 2021; Song and Yang, 2024).

These findings from mouse models indicate that hyperoxia alters the gut microbiome, marked by a reduction in SCFA-producing OA and an increase in FA, particularly *Enterobacteriaceae*, which is commonly associated with dysbiosis. The depletion of OA appears to precede the expansion of *Enterobacteriaceae* (Li H. et al., 2021; Li Y. et al., 2021), suggesting that depletion of butyrate plays a crucial role in hyperoxia-induced gut dysbiosis. However, these murine studies have limitations, as they expose healthy animals to nearly pure inhaled O_2_, a condition rarely encountered in clinical settings, especially in patients without hypoxia (O’Driscoll et al., 2017; Siemieniuk et al., 2018).

## Hyperoxia during VA-ECMO disrupts gut homeostasis via metabolic reprogramming and dysbiosis

Under peripheral VA-ECMO support, the typical hemodynamic pattern involves a competitive flow between blood pumped by the heart and blood flowing retrograde through the aorta from the ECMO reinfusion cannula in the femoral artery (Asija et al., 2023). This leads to overproduction of ROS and inhibits the growth of OA, thereby suppressing butyrate expression and shifting colonocyte metabolism from oxidative phosphorylation to fermentation. The metabolic shift reduces the consumption of O_2_ by colonocytes, leading to further accumulation of luminal O_2_ and exacerbating the disruption of anaerobiosis. The increased availability of O_2_ as exogenous electron receptors finally promotes the growth of FA, such as *Enterobacteriaceae*, causing dysbiosis (Figure 4C). PPAR, a nuclear receptor transcription factor, plays a key role in regulating cellular energy metabolism and mitochondrial function. Among its subtypes, PPAR-*γ* is highly expressed in colon cells. Studies, primarily in cellular and murine models, have shown that butyrate, as a natural ligand of PPAR-γ, can directly activate this receptor to promote mitochondrial oxidative phosphorylation and suppress anaerobic glycolysis (Byndloss et al., 2017). On the other hand, HIF-1α, as an oxygen-sensitive transcription factor, plays a central role in cellular adaptation to hypoxia and the regulation of glycolysis (Semenza, 2012). High expression of HIF-1α reprograms cellular energy metabolism from aerobic respiration to anaerobic glycolysis (Lin et al., 2023). Notably, PPAR-γ activation inhibits the HIF-1α signaling pathway, creating an important antagonistic relationship in metabolic regulation (Blum et al., 2016) (Figure 4D). Furthermore, research in murine models has established that physiological hypoxia in the gut is essential for maintaining host-microbiota balance. Short-chain fatty acids (such as butyrate) produced by microbial metabolism of dietary fibers enhance mitochondrial respiration in intestinal epithelial cells, thereby consuming oxygen and subsequently stabilizing and activating HIF-1α. Stabilized HIF-1α is critical for reinforcing epithelial barrier function and regulating IL-22 production by ILC3s (Pral et al., 2021). Conversely, hyperoxia disrupts this hypoxic microenvironment, not only inhibiting the HIF-1 signaling pathway and impairing barrier function but also promoting the expansion of FA, thereby serving as a key driver of gut microbiota dysbiosis and inflammation.

## Current perspectives and future challenges

From an ecological perspective, the dynamic changes associated with gut dysbiosis are primarily driven by dysfunction of the gut barrier, which allows O_2_ to leak into the lumen and creates an aerobic nutrient niche that suppresses butyrate-productive OA and favors the growth of pathogenic FA. The central role of O_2_ in gut dysbiosis has been indirectly demonstrated in various human diseases, where O_2_ acts as an intermediate factor. Recently, emerging evidence suggests that hyperoxia during O_2_ therapy can directly drive gut dysbiosis, with O_2_ serving as the initiating factor. In clinical settings, hyperoxia during VA-ECMO oxygenates the patient from the “bottom up,” potentially raising O_2_ levels in the intestinal lumen and disrupting the redox balance between obligate and FA—ultimately contributing to dysbiosis. Further clinical and basic research is needed to better understand how hyperoxia during O_2_ therapy affects redox dynamics in intestinal microbial ecology, and to identify optimal oxygenation strategies for patients undergoing VA-ECMO support.

It is crucial to recognize, however, that gut dysbiosis in critically ill patients, including those on VA-ECMO, is a multifactorial phenomenon. Beyond hyperoxia, factors such as altered enteral nutrition, profound physical inactivity, physiological stress, and the use of broad-spectrum antibiotics are known to independently contribute to microbial imbalance. Nevertheless, this article specifically highlights that hyperoxia remains a potentially critical and underappreciated driver in this context, owing to its direct inhibitory effect on OA. The unique iatrogenic hyperoxemia experienced by VA-ECMO patients may thus represent a major and persistent insult that amplifies the dysbiotic effects of other factors.

Indeed, alterations in the oxygen microenvironment play a critical role in various gut disorders associated with dysbiosis. Taking *Clostridium difficile* infection as an example, the normal hypoxic gut environment favors the survival of OA, which form a biological barrier inhibiting the colonization of *C. difficile*. When factors such as antibiotics induce dysbiosis, the hypoxic gut environment is disrupted, weakening the protective role of anaerobic bacteria and potentially promoting the proliferation of *C. difficile* spores, thereby significantly increasing host susceptibility (Khazaaleh et al., 2022). In summary, disturbances in intestinal oxygen balance—whether hypoxia or hyperoxia—are key mechanisms driving the onset and progression of disease.

Translating findings on oxygen and gut microbiota from animal and cellular studies to humans still poses significant challenges. Oxygen, the host, and the microbial community form a complex multi-factorial structure, making it particularly challenging to establish similar causal relationships in humans. Factors such as ethical constraints in human studies, variations in microbial composition and function, and confounding variables like diet and antibiotic use further complicate research. Nevertheless, animal models have provided strong evidence for hyperoxia-induced dysbiosis (Xing et al., 2020; Chen et al., 2023). However, direct clinical evidence linking hyperoxia to changes in gut microbiota remains relatively scarce. This is particularly pronounced in critically ill patients—where confounding factors such as underlying diseases, antibiotic use, and enteral nutrition are abundant, and longitudinal collection of intestinal samples presents practical challenges. To address this evidence gap, future clinical studies should prioritize specific populations, such as patients receiving veno-arterial extracorporeal membrane oxygenation (VA-ECMO) who are exposed to severe lower-body hyperoxia (Dai et al., 2024). Furthermore, prospective studies should be conducted within this population to analyze the correlation between post-oxygenator arterial oxygen partial pressure (P_POST_O_2_) and serially measured values of gut injury markers (such as intestinal fatty acid-binding protein), microbial composition, and metabolic profiles. This approach is expected to yield more direct evidence. Therefore, future research should adopt more comprehensive and longitudinal methods, integrating multi-omics data such as metagenomics and transcriptomics, to advance the field from correlation studies toward clinical translation.

It must be acknowledged that this review has certain limitations. First, we have highlighted the challenges in translating findings from animal models to humans, emphasizing that while animal models are indispensable for mechanistic studies, there are significant differences in gut microbiota across species. Therefore, the primary focus of this paper is not to directly predict clinical outcomes but to propose, for the first time, the novel concept that “hyperoxia is a driver of gut microbiota dysbiosis in critically ill patients, “aiming to establish a theoretical foundation for future targeted human studies. Second, we recognize that current clinical evidence is largely derived from observational studies, which are highly susceptible to confounding factors such as antibiotic use and nutritional support, making it difficult to establish a pure causal relationship between hyperoxia and microbiota dysbiosis. Despite these limitations, the conceptual framework proposed in this paper provides a solid foundation for subsequent research. Future work should focus on designing more rigorous prospective studies or mechanistic explorations to isolate and quantify the independent effects of hyperoxia on gut microbiota under controlled conditions.

## Glossary

VA-ECMO: Venoarterial-extracorporeal membrane oxygenation
VV-ECMO: Venovenous-extracorporeal membrane oxygenation
CS: Cardiogenic shock
CA: Cardiac arrest
ATP: Adenosine triphosphate
PO_2_: Oxygen partial pressure
P_POST_O_2_: Post-oxygenator oxygen partial pressure
SCFA: Short chain fatty acid
ROS: Reactive oxygen species
SOD: Superoxide dismutase
Ga: Giga-annum
GOE: Great oxygenation event
FA: Facultative anaerobes
OA: Obligate anaerobes
NADH: Nicotinamide adenine dinucleotide
DO_2_: O_2_ delivery
VO_2_: O_2_ consumption
CO: Cardiac output
CaO_2_: Arterial O_2_ content
MODS: Multiple organ dysfunction syndrome
SaO_2_: O_2_ saturation
ARDS: Acute respiratory distress syndrome
FiO_2_: Fraction of inspired O_2_
P_A_O_2_: Partial pressure of alveolar O_2_
PvO_2_: Venous O_2_ pressure
FbO_2_: fraction of inspired O_2_ of blender
PPAR-*γ*: Peroxisome proliferator–activated receptor gamma
HIF-1α: Hypoxia-inducible factor 1-Alpha
